# Development and validation of a prognostic nomogram to predict 30-day all-cause mortality in patients with CRO infection treated with colistin sulfate

**DOI:** 10.3389/fphar.2024.1409998

**Published:** 2024-07-17

**Authors:** Wei Li, Yu Liu, Lu Xiao, Xuezhou Cai, Weixi Gao, Dong Xu, Shishi Han, Yan He

**Affiliations:** ^1^ Department of Pharmacy, Tongji Hospital, Tongji Medical College, Huazhong University of Science and Technology, Wuhan, China; ^2^ Department of Rehabilitation, Tongji Hospital, Tongji Medical College, Huazhong University of Science and Technology, Wuhan, China; ^3^ Department of Pharmacy, Xianning Central Hospital, Hubei University of Science and Technology, Xianning, China; ^4^ Department of Pharmacy, Renmin Hospital of Wuhan University, Wuhan, China; ^5^ Department of Infection Disease, Tongji Hospital, Tongji Medical College, Huazhong University of Science and Technology, Wuhan, China; ^6^ Yichang Health Technology Information Center, Yichang, China

**Keywords:** carbapenem-resistant gram-negative organisms, colistin sulfate, 30-day mortality, prediction nomogram, CRO

## Abstract

**Background:**

Carbapenem-resistant Gram-negative organism (CRO) infection is a critical clinical disease with high mortality rates. The 30-day mortality rate following antibiotic treatment serves as a benchmark for assessing the quality of care. Colistin sulfate is currently considered the last resort therapy against infections caused by CRO. Nevertheless, there is a scarcity of reliable tools for personalized prognosis of CRO infections. This study aimed to develop and validate a nomogram to predict the 30-day all-cause mortality in patients with CRO infection who underwent colistin sulfate treatment.

**Methods:**

A prediction model was developed and preliminarily validated using CRO-infected patients treated with colistin sulfate at Tongji Hospital in Wuhan, China, who were hospitalized between May 2018 and May 2023, forming the study cohort. Patients admitted to Xianning Central Hospital in Xianning, China, between May 2018 and May 2023 were considered for external validation. Multivariate logistic regression was performed to identify independent predictors and establish a nomogram to predict the occurrence of 30-day all-cause mortality. The receiver operating characteristic (ROC) curve, the area under the ROC curve (AUC), and the calibration curve were used to evaluate model performance. The decision curve analysis (DCA) was used to assess the model clinical utility.

**Results:**

A total of 170 patients in the study cohort and 65 patients in the external validation cohort were included. Factors such as age, duration of combination therapy, nasogastric tube placement, history of previous surgery, presence of polymicrobial infections, and occurrence of septic shock were independently associated with 30-day all-cause mortality and were used to construct the nomogram. The AUC of the nomogram constructed from the above six factors was 0.888 in the training set. The Hosmer-Lemeshow test showed that the model was a good fit (*p* = 0.944). The calibration curve of the nomogram was close to the ideal diagonal line. Furthermore, the decision curve analysis demonstrated significantly better net benefit in the model. The external validation proved the reliability of the prediction nomogram.

**Conclusion:**

A nomogram was developed and validated to predict the occurrence of 30-day all-cause mortality in patients with CRO infection treated with colistin sulfate. This nomogram offers healthcare providers a precise and efficient means for early prediction, treatment management, and patient notification in cases of CRO infection treated with colistin sulfate.

## Introduction

Infections caused by carbapenem-resistant Gram-negative organisms (CROs), including carbapenem-resistant *Acinetobacter baumannii* (CRAB), carbapenem-resistant *Pseudomonas aeruginosa* (CRPA) and carbapenem-resistant Enterobacteriaceae (CRE), have attracted widespread attention due to their rapid growth, treatment challenges, high mortality and heavy economic burden (Gutiérrez-Gutiérrez et al., 2016; Tamma et al., 2017a). It is estimated that up to two million patients in the United States experience antibiotic-resistant bacterial infections (Gram-positive and/or Gram-negative) annually, leading to over 23,000 deaths, with a significant portion attributed to CRO infections (Centers for Disease Control and Prevention, 2024). Some recent evidence has highlighted increased mortality in patients with CROs compared with that of carbapenem-susceptible counterparts (Kadri et al., 2019; Li et al., 2020; Maraolo et al., 2021; Zhou et al., 2021; Hoo et al., 2022; Paniagua-García et al., 2024). In addition, the alarming increase in carbapenem resistance and the limited availability of antimicrobial options undoubtedly contribute to the mortality rate. Effective management of CRO infections presents a significant medical challenge (Veeraraghavan et al., 2019).

Polymyxin-based combination therapy has become the last-line treatment for CRO infections (Veeraraghavan et al., 2019; Hao et al., 2022). In China, three forms of polymyxins are currently in clinical use: colistimethate sodium, polymyxin B sulfate, and colistin sulfate. Colistin sulfate, developed independently in China by the Institute of Antibiotics, Chinese Academy of Medical Sciences, was reintroduced to the market in 2019. Unfortunately, crude mortality rates of greater than 40% are still occasionally reported for CRO infections treated with polymyxin B or colistimethate sodium treatments (Falagas et al., 2021; Wang et al., 2023). And even though treatment with these two polymyxins could achieve a good clinical response, some studies did not find a reduction in all-cause mortality at Day 28 or Day 30, either with monotherapy or in combination (Durante-Mangoni et al., 2013; Lodise et al., 2022). The observed 28-day or in-hospital all-cause mortality rates also varied from studies for colistin sulfate, with a wide range of 9.3%–44% (Hao et al., 2022; Jin et al., 2022; Lu et al., 2022; Peng et al., 2023). However, compared with colistimethate sodium or polymyxin B sulfate, colistin sulfate is associated with less nephrotoxicity, as demonstrated in recent real-world studies (Hao et al., 2022; Jin et al., 2022; Lu et al., 2022; Wang et al., 2022; Peng et al., 2023). These findings suggest the necessity for further evaluation of the mortality risk factors related to CRO infections during colistin sulfate treatment.

To our knowledge, no predictive model has yet been constructed to evaluate the impact of relevant factors on mortality in CRO-infected patients receiving colistin sulfate-based regimens. Early assessment of factors and prediction of associated outcomes will undoubtedly be meaningful to clinicians, providing the opportunity to adjust regimens and improve patient survival. This will aid healthcare providers in the early identification of patient treatment responses, enabling the selection of more rational treatment regimens and alleviating the financial burden on patients. Therefore, it is of utmost importance to enable accurate and rapid tools to predict the prognosis and survival of CRO infections treated with colistin sulfate. Here, this study aims to identify risk factors associated with all-cause mortality in CRO infections in patients undergoing treatment with a colistin sulfate-based regimen. Additionally, a nomogram will be constructed and validated to provide a reference tool for risk assessment.

## Methods

### Study design and population

This study was designed as a retrospective investigation of the data from patients with CRO infections and treated with colistin sulfate. The study population consisted of patients from Tongji Hospital, Tongji Medical College, Huazhong University of Science and Technology, Wuhan, China for model construction and preliminary validation, and patients from Xianning Central Hospital, Xianning, China, for external validation. The study protocol was approved by the Ethics Committees of both hospitals [Ethical Approval Code: TJ-IRB202308134 and XYLZ-Y (2022)011] and with informed consent waived due to the retrospective nature of the study.

The study cohort included patients with CRO infections and treated with colistin sulfate who were admitted to Tongji Hospital between May 2018 and May 2023. These enrolled patients were randomly assigned to either the training or validation set in an approximately 7:3 ratio. In addition, another independent cohort of patients from Xianning Central Hospital admitted between May 2018 and May 2023 were also enrolled as an external validation cohort. Both hospitals enrolled patients based on the same criteria.

Diagnosis of CRO Infection: 1) If the patient’s pathogen-positive specimens are from sputum, throat swabs, midstream urine, feces, etc., and the same type of CRO strain is cultured ≥2 times from the same or different specimens, then a CRO infection is confirmed. If only one CRO strain is cultured, and clinically accompanied by any of the following signs of infection or radiological evidence, then a CRO infection is confirmed: a) body temperature >38°C or <36°C; b) chills, shortness of breath, or hypotension (excluding other related possibilities); c) presence of pyuria, cloudy urine, purulent sputum, redness, swelling, heat, pain in skin mucous membranes, or rupture on the body surface with purulent secretion; d) pulmonary imaging suggests inflammatory changes in the lungs; e) X-ray or CT indicates serous cavity effusion or abscess (pleural effusion, ascites, liver abscess, renal abscess, etc.) (Chen et al., 2021). Treatment options for CRO patients are colistin based, combined with tigecycline, fosfomycin, carbapenems or aminoglycosides. Patients meeting the following criteria were included: ① diagnosed with CRO infections, ② treated with colistin sulfate and ③ had complete medical records. Cases with a colistin sulfate treatment duration of less than 3 days or patients under 18 years of age were excluded from the study.

### Data collection

All research data were collected from patients diagnosed with CRO infections and treated with colistin sulfate at a daily dosage of 1–1.5 million units, administered 2–3 times per day. The following clinical information was collected: age, gender, presence of acute or chronic comorbidities (i.e., diabetes, COPD, cancer, chronic hepatitis, chronic kidney disease, HIV, neutropenia, and solid organ transplantation), Charlson’s Comorbidity Index (Charlson et al., 1987)) and APACHE II score, history of previous surgery, use of steroid and/or immunosuppressive therapy (≤30 days before onset), and any invasive procedures (≤72 h before onset). The isolation of CRO strains from other sites (≤30 days) or co-infections was also considered. Sepsis or septic shock were evaluated according to the criteria of the International Consensus Definition for Sepsis and Septic Shock (Singer et al., 2016). Treatment variables included antibiotic therapy with colistin sulfate in either monotherapy or combination therapy, the durations of antibiotic therapy and combination therapy, as well as the use of premedication.

### Definitions

The diagnosis of an infection is mainly based on the patient’s laboratory test results, microbial culture findings, and the expertise of the clinician. Comorbidities included diabetes mellitus, chronic obstructive pulmonary disease, hematological neoplasms, solid tumors, chronic hepatitis, cardiovascular disease, nervous system disease, chronic kidney disease, HIV, neutropenia, endocrine system disease, gastrointestinal disease, organ transplantation, and other conditions.

All CRO infections were identified in the microbiology laboratory. The CROs detected included *Klebsiella pneumoniae*, *Pseudomonas aeruginosa*, *Escherichia coli*, and *A. baumannii*. Bacterial identification and susceptibility testing were performed using the Vitek^®^2 automated system (Biomerieux, France). Sensitivity was interpreted according to the criteria of the Clinical and Laboratory Standards Institute. Enterobacteriaceae with a minimum inhibitory concentration (MIC) ≥4 μg/mL were considered to be carbapenem-resistant. *Pseudomonas aeruginosa* and *Acinetobacter* were considered to be carbapenem-resistant if their MIC was ≥8 μg/mL. The clinicians assessed the pathogenicity of the pathogens according to the distribution characteristics of pathogens in the institution and their own professional experience.

### Development and assessment of the nomogram

A multivariate logistic regression analysis was used to construct a nomogram model to predict the occurrence of mortality. Independent predictors (*p* < 0.05) identified through the multivariate logistic regression were utilized to construct the nomogram. The nomogram allowed for the visualization of predictor lines moving upwards to determine the corresponding points. The cumulative points were then located on the “Total Points” axis, and a line was drawn downwards to intersect with the lower scales, providing the probability of mortality. Thereafter, the external validation of the visual prediction model was conducted. The receiver operating characteristic (ROC) curve, the area under the ROC curve (AUC), and the calibration curve were used to evaluate the predictive accuracy and conformity of the model. The decision curve analysis (DCA) reflected the net benefit of the model for patients. Both discrimination and calibration were assessed by bootstrapping with 1,000 resamples.

### Statistical analysis

All statistical analyses were performed using the R statistical software, version 4.0.3 (R Foundation for Statistical Computing; statistical https://www.r-project.org/). Logistic regression algorithm and nomogram construction were conducted using the “rms” package. The ROC curve was plotted using the “pROC” package. The Hosmer-Lemeshow test was performed using the “vcdExtra” package. DCA was performed with the “dca.R” function. All statistical tests were two-tailed, and *p* < 0.05 were considered statistically significant.

## Results

### General characteristics

**FIGURE 1:**
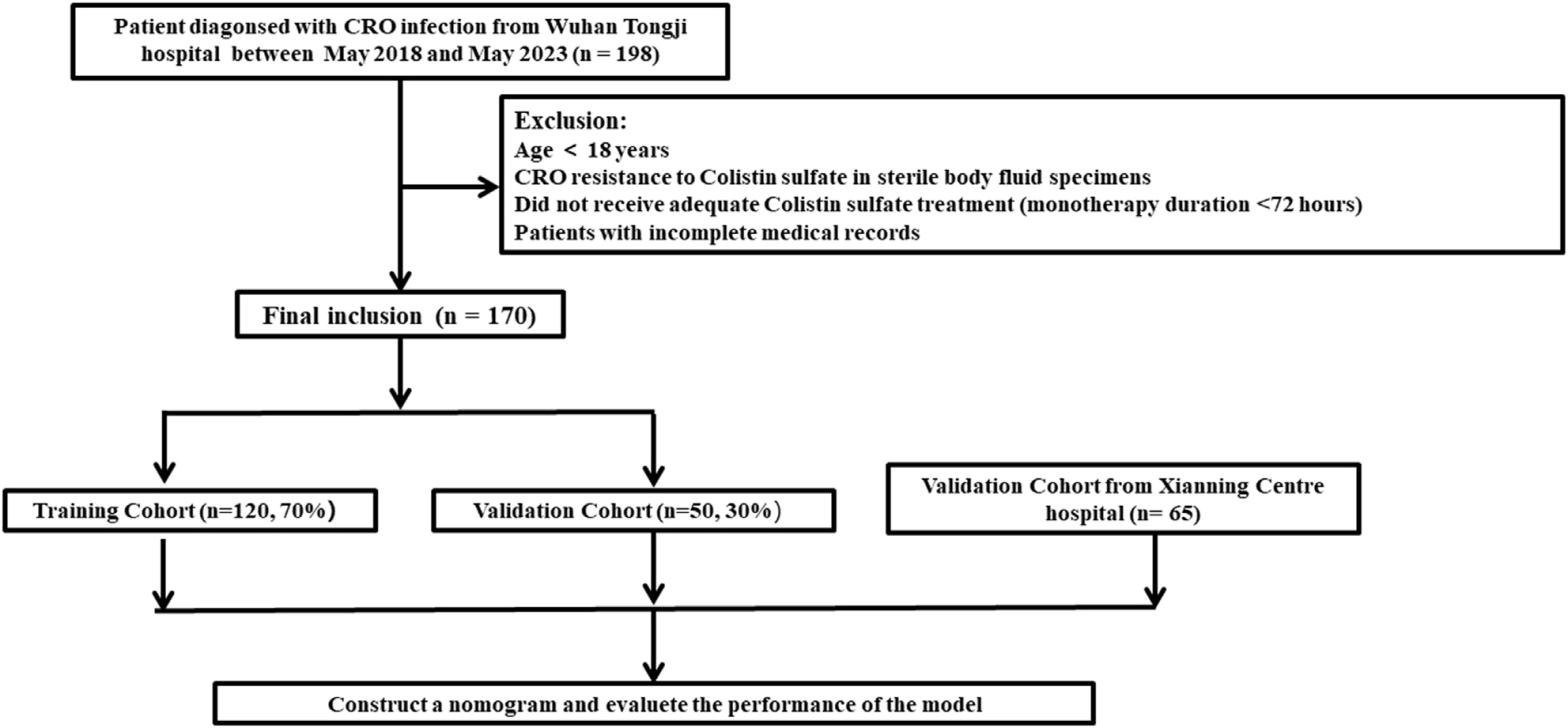
Flow chart for patient selection.

The clinical data for a total of 198 patients was obtained from Tongji Hospital, Huazhong University of Science and Technology. Of these, 28 patients did not meet the inclusion criteria. Consequently, the study cohort consisted of 170 patients (Figure 1). Among them, 120 patients were allocated to the training set while the remaining 50 patients were allocated to the validation set for modeling and preliminary validation, respectively. An external cohort of 65 patients from Xianning Central Hospital was used to verify the performance of the model. The characteristics of the patients included in this study are summarized in Table 1. All enrolled patients received colistin sulfate-based treatment. The majority were male with a median age of 49 years. Most of the patients presented comorbidity, with the most frequent being cardiovascular, followed by hepatic and renal diseases or hematological malignancies. Charlson Comorbidity Index ≥3 was found in 54.1% and 43.1% of patients in the study cohort and external validation cohort, respectively. The most frequently isolated pathogen was *K. pneumoniae KPC*, followed by *A. baumannii*, *P. aeruginosa,* and *E. coli*. The incidence of all-cause mortality in the two sets was well comparable with rates of 29.2% and 30.0% in the training and validation sets in the study cohort, respectively (*p* = 0.413). There were no significant differences in all clinical factors between the training and validation sets, with *p*-values ranging from 0.171 to 1.000 (Table 1). There were no significant differences between the external validation group and the training group in the variables except for the duration of aerosolized colistin sulfate, presence of solid tumor, nasogastric tube use, hormone therapy, multiple infections, pneumonia, premedication, and combination medication (Table 1).

**TABLE 1:** Baseline characteristics of all patients in the study and external validation cohorts.

Variables		Study cohort				External validation cohort	
		All (n = 170)	Validation set (n = 50)	Training set (n = 120)	*p*-value	All (n = 65)	*p*-value
Sex	Male	116 (68.2%)	34 (68.0%)	82 (68.3%)	1.000	40 (61.5%)	0.442
	Female	54 (31.8%)	16 (32.0%)	38 (31.7%)		25 (38.5%)	
Age (years)	<40	37 (21.8%)	8 (16.0%)	29 (24.2%)	0.085	14 (21.5%)	0.594
	40–60	83 (48.8%)	31 (62.0%)	52 (43.3%)		25 (38.5%)	
	>60	50 (29.4%)	11 (22.0%)	39 (32.5%)		26 (40.0%)	
Drug combination	carbapenem	82 (48.2%)	25 (50.0%)	57 (47.5%)	0.898	35 (53.8%)	0.503
	Beta-lactamase inhibitors	68 (40.0%)	19 (38.0%)	49 (40.8%)	0.864	20 (30.8%)	0.233
	colistin sulfate atomization	17 (10.0%)	4 (8.00%)	13 (10.8%)	0.779	26 (40.0%)	<0.001
	Tigecycline	47 (27.6%)	13 (26.0%)	34 (28.3%)	0.903	16 (24.6%)	0.711
Charlson’s Comorbidity Index	≥3	92 (54.1%)	27 (54.0%)	65 (54.2%)	1.000	28 (43.1%)	0.523
Wards submitting index culture	Intensive care unit	9 (5.29%)	3 (6.00%)	6 (5.00%)	1.000	0 (0.00%)	0.190
	Surgery	14 (8.24%)	4 (8.00%)	10 (8.33%)		8 (12.3%)	
	Medicine	49 (28.8%)	14 (28.0%)	35 (29.2%)		17 (26.2%)	
	Organ transplantation	98 (57.6%)	29 (58.0%)	69 (57.5%)		39 (60.0%)	
Comorbidities	Diabetes	21 (12.4%)	6 (12.0%)	15 (12.5%)	1.000	10 (15.4%)	0.747
	Hematological malignancies	50 (29.4%)	11 (22.0%)	39 (32.5%)	0.236	22 (33.8%)	0.982
	Solid tumors	17 (10.0%)	3 (6.00%)	14 (11.7%)	0.400	1 (1.54%)	0.033
	Chronic Hepatitis	65 (38.2%)	21 (42.0%)	44 (36.7%)	0.632	26 (40.0%)	0.774
	Cardiovascular Disease	88 (51.8%)	29 (58.0%)	59 (49.2%)	0.378	28 (43.1%)	0.523
	Chronic kidney disease	52 (30.6%)	16 (32.0%)	36 (30.0%)	0.940	21 (32.3%)	0.875
	Neutropenia	31 (18.2%)	7 (14.0%)	24 (20.0%)	0.481	18 (27.7%)	0.313
	Gastrointestinal disease	27 (15.9%)	9 (18.0%)	18 (15.0%)	0.797	14 (21.5%)	0.358
eGFR (mL/min)	>90	95 (55.9%)	30 (60.0%)	65 (54.2%)	0.948	36 (55.4%)	0.175
	60–90	37 (21.8%)	10 (20.0%)	27 (22.5%)		7 (10.8%)	
	30–60	27 (15.9%)	7 (14.0%)	20 (16.7%)		15 (23.1%)	
	<30	11 (6.47%)	3 (6.00%)	8 (6.67%)		7 (10.8%)	
Pre-infection variables	Central venous catheter	129 (75.9%)	41 (82.0%)	88 (73.3%)	0.314	34 (52.3%)	0.007
	Nasogastric tube	73 (42.9%)	26 (52.0%)	47 (39.2%)	0.171	44 (67.7%)	<0.001
	Surgical drainage	91 (53.5%)	28 (56.0%)	63 (52.5%)	0.804	26 (40.0%)	0.141
	Bladder catheter	49 (28.8%)	15 (30.0%)	34 (28.3%)	0.974	16 (24.6%)	0.711
	Steroid therapy	74 (43.5%)	24 (48.0%)	50 (41.7%)	0.556	8 (12.34%)	<0.001
	Immunosuppressive therapy	47 (27.6%)	12 (24.0%)	35 (29.2%)	0.618	18 (27.7%)	0.967
	Previous surgery	66 (38.8%)	22 (44.0%)	44 (36.7%)	0.471	15 (23.1%)	0.084
Infection variables	Polymicrobial infections	24 (14.1%)	7 (14.0%)	17 (14.2%)	1.000	26 (40.0%)	<0.001
	Septic shock	54 (31.8%)	17 (34.0%)	37 (30.8%)	0.823	21 (32.3%)	0.968
	Pneumoniae	130 (76.5%)	37 (74.0%)	93 (77.5%)	0.770	40 (61.5%)	0.033
	Bloodstream infections	64 (62.35%)	19 (38.0%)	45 (37.5)	0.951	5 (7.69%)	<0.001
Pathogens	*K. pneumoniae* KPC	75 (44.1%)	25 (50.0%)	50 (41.7%)	0.408	22 (33.8%)	0.377
	*P. aeruginosa*	23 (13.5%)	9 (18.0%)	14 (11.7%)	0.393	13 (20.0%)	0.189
	*E. coli*	10 (5.88%)	1 (2.00%)	9 (7.50%)	0.284	3 (4.62%)	0.545
	*Acinetobacter* baumannii	38 (22.4%)	12 (24.0%)	26 (21.7%)	0.896	16 (24.6%)	0.785
Treatment variables	premedication	52 (30.6%)	14 (28.0%)	38 (31.7%)	0.772	32 (49.2%)	0.028
Days of antibiotic therapy	<7	82 (48.2%)	26 (52.0%)	56 (46.7%)	0.814	26 (40.0%)	0.265
	7–14	63 (37.1%)	17 (34.0%)	46 (38.3%)		28 (43.1%)	
	>14	25 (14.7%)	7 (14.0%)	18 (15.0%)		9 (13.8%)	
Days of combination therapy	≤3	23 (13.5%)	7 (14.0%)	16 (13.3%)	0.741	27 (41.5%)	<0.001
	4–7	68 (40.0%)	22 (44.0%)	46 (38.3%)		22 (33.8%)	
	>7	79 (46.5%)	21 (42.0%)	58 (48.3%)		16 (24.6%)	

### Screening for predictive factors

Univariate analysis was used to screen variables, and variables achieving a statistically significant level of *p* < 0.1 were included in the multivariate logistic regression analysis to further identify independent predictors. Fifteen candidate variables, including urinalysis age, Charlson’s Comorbidity Index, diabetes, hematological malignancies, chronic kidney disease, nasogastric tube, bladder catheter, pneumoniae, cardiovascular disease, duration of combination therapy, neutropenia, eGFR, previous surgery, polymicrobial infections, septic shock (all *p* < 0.1), were significantly associated with mortality in the univariate logistic regression analyses (Table 2). Among these, age (40–60 years) (*p* = 0.030), nasogastric tube (*p* = 0.002), previous surgery (*p* = 0.021), septic shock (*p* = 0.010), days of combination therapy (>7 days) (*p* = 0.023) and polymicrobial infections (*p* = 0.039), were identified as independent risk factors for mortality by the subsequent multivariate regression analysis (Table 2).

**TABLE 2:** Univariate and multivariate logistic regression analysis for patients in the training set.

Variables		Univariate analysis		Multivariate analysis	
		Or (95% CI)	*p*-value	Or (95% CI)	*p*-value
Sex	Male				
	Female	0.916 (0.373–2.146)	0.843	–	–
Age (years)	<40				
	40–60	3.193 (0.928–14.85)	0.09	12.77 (1.573–170.3)	0.030
	>60	6.029 (1.737–28.34)	0.009	8.462 (0.898–109.4)	0.076
Drug combination	Carbapenem	0.635 (0.276–1.424)	0.275	–	–
	Beta-lactamase inhibitors	0.645 (0.271–1.469)	0.305	–	–
	Colistin sulfate atomization	1.195 (0.305–3.984)	0.78	–	–
	Tigecycline	1.391 (0.572–3.279)	0.455	–	–
Charlson’s Comorbidity Index	≥3	3.258 (1.415–7.949)	0.007	1.68 (0.353–8.583)	0.515
Wards submitting index culture	Intensive care unit				
	Surgery	4.667 (0.58–50.86)	0.164	–	–
	Medicine	0.8 (0.133–6.429)	0.813	–	–
	Organ transplantation	0.509 (0.089–3.936)	0.461	–	–
Comorbidities	Diabetes	2.659 (0.857–8.123)	0.083	1.243 (0.085–16.67)	0.871
	Hematological malignancies	0.205 (0.057–0.577)	0.006	0.529 (0.034–6.558)	0.627
	Solid tumors	1.548 (0.444–4.887)	0.466	–	–
	Chronic Hepatitis	1.667 (0.731–3.784)	0.221	–	–
	Cardiovascular Disease	2.257 (1.001–5.278)	0.054	0.741 (0.338–1.623)	0.452
	Chronic kidney disease	3.803 (1.633–9.038)	0.002	5.774 (1.052–40.60)	0.055
	Neutropenia	0.314 (0.07–1.002)	0.077	0.369 (0.027–4.782)	0.442
	Gastrointestinal disease	1.86 (0.627–5.242)	0.245	–	–
eGFR (mL/min)	>90				
	60–90	1.684 (0.587–4.663)	0.319	0.783 (0.104–5.719)	0.807
	30–60	2.667 (0.887–7.894)	0.075	2.595 (0.42–16.85)	0.301
	<30	4 (0.847–19.09)	0.073	3.821 (0.219–80.56)	0.366
Pre-infection variables	Central venous catheter	1.19 (0.485–3.145)	0.712	–	–
	Nasogastric tube	4.106 (1.792–9.803)	0.001	19.97 (3.617–178.8)	0.002
	Surgical drainage	1.875 (0.832–4.376)	0.135	–	–
	Bladder catheter	3.608 (1.536–8.611)	0.003	0.788 (0.124–4.472)	0.791
	Steroid therapy	0.615 (0.259–1.401)	0.256	–	–
	Immunosuppressive therapy	0.711 (0.271–1.727)	0.466	–	–
	Previous surgery	0.453 (0.174–1.083)	0.086	0.149 (0.025–0.679)	0.021
Infection variables	Polymicrobial infections	2.773 (0.949–8.028)	0.058	9.937 (1.216–104.4)	0.039
	Septic shock	2.05 (0.88–4.754)	0.093	9.17 (1.931–59.82)	0.01
	Pneumoniae	3.81 (1.208–16.91)	0.040	4.387 (0.635–39.20)	0.152
	Bloodstream infections	0.646 (0.265–1.493)	0.318	–	–
Pathogens	*K. pneumoniae* KPC	1.238 (0.547–2.781)	0.604	–	–
	*P. aeruginosa*	1.548 (0.444–4.887)	0.466	–	–
	*E. coli*	1.35 (0.272–5.465)	0.685	–	–
	*Acinetobacter* baumannii	1.227 (0.455–3.106)	0.673	–	–
Treatment variables	premedication	0.75 (0.297–1.778)	0.525	–	–
Days of antibiotic therapy	<7				
	7–14	0.635 (0.265–1.48)	0.299	4.083 (0.256–76.33)	0.321
	>14	0.106 (0.006–0.577)	0.035	0.11 (0.001–7.743)	0.347
Days of combination therapy	≤3				
	4–7	0.376 (0.113–1.196)	0.1	0.489 (0.05–4.355)	0.517
	>7	0.143 (0.04–0.473)	0.002	0.032 (0.001–0.504)	0.023

### Risk prediction nomogram development

**FIGURE 2:**
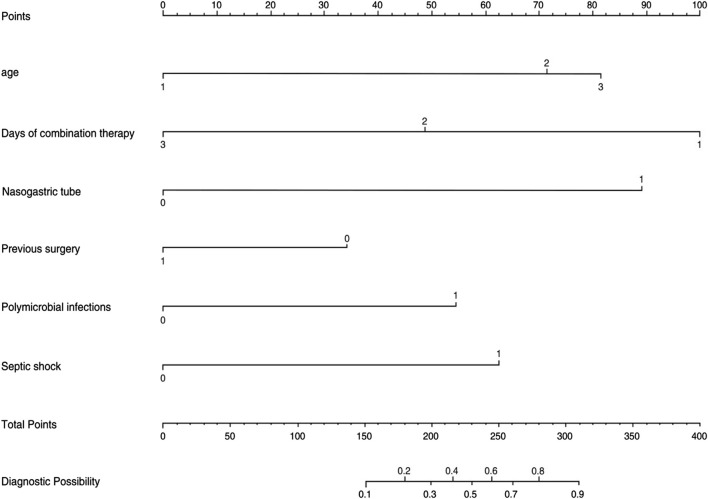
Nomogram for the perinatal prediction of 30-day mortality in patients with CRO infections.

The logistic regression model was constructed based on the six factors (Table 3) identified above. Subsequently, these infectious factors were integrated into the nomogram (Figure 2). For each patient, higher total points indicated a higher risk of mortality. In addition, the Hosmer−Lemeshow test demonstrated that the model fits well (*p* = 0.601).

**TABLE 3:** The logistic regression of six perinatal factors used for constructing model.

Variables	β	SE	Z	*p*-value	OR	Or 95% CI	
						L	U
Age (years)							
40–60	1.981	0.8552	2.316	0.021	7.247	1.551	46.81
>60	2.257	0.89251	2.529	0.011	9.553	1.886	66.19
Days of combination therapy							
4–7	−1.42	0.84528	−1.679	0.093	0.242	0.042	1.209
>7	−2.772	0.88223	−3.142	0.002	0.063	0.01	0.321
Nasogastric tube	2.469	0.64732	3.814	0.000	11.811	3.631	47.54
Previous surgery	−0.947	0.5772	−1.641	0.101	0.388	0.118	1.159
Polymicrobial infections	1.513	0.77882	1.943	0.052	4.54	0.979	21.81
Septic shock	1.734	0.60978	2.844	0.004	5.664	1.806	20.3

SE, standard error; OR, odds ratio; CI, confidence interval; L, lower limit; U, upper limit.

### Predictive accuracy and net benefit of the nomogram

**FIGURE 3:**
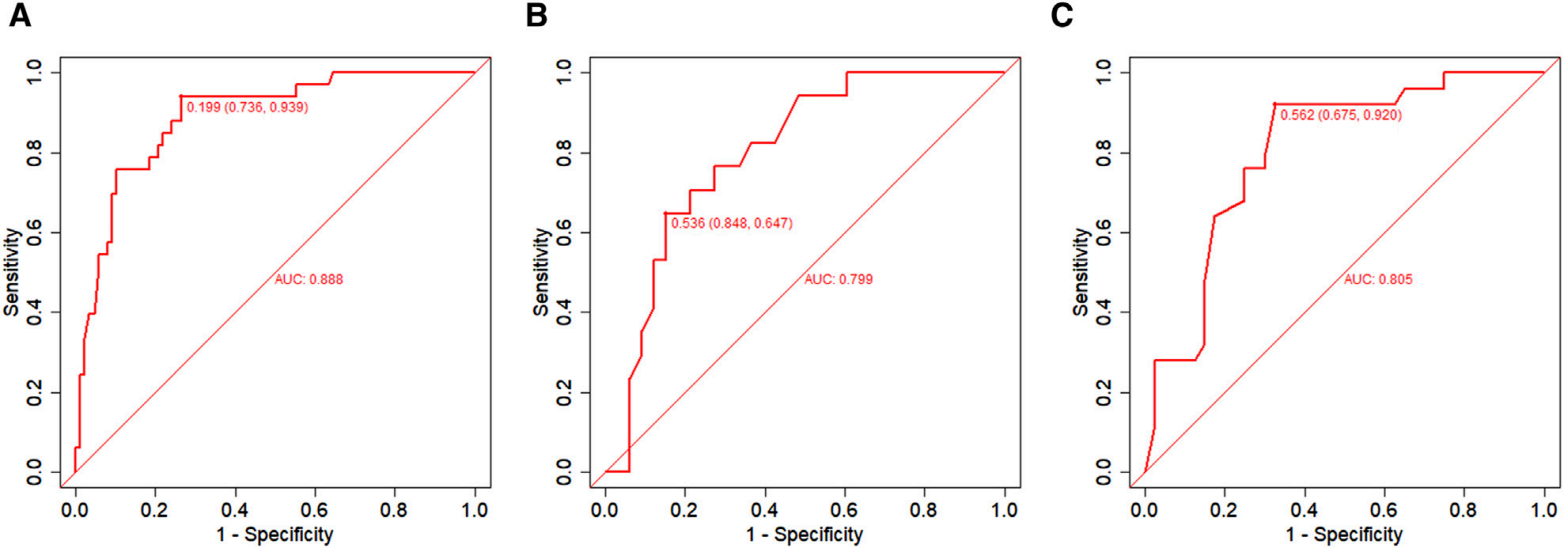
ROC curves. **(A)** Training set; **(B)** Validation set; **(C)** External validation cohort. AUC = area under the ROC curve.

**FIGURE 4:**
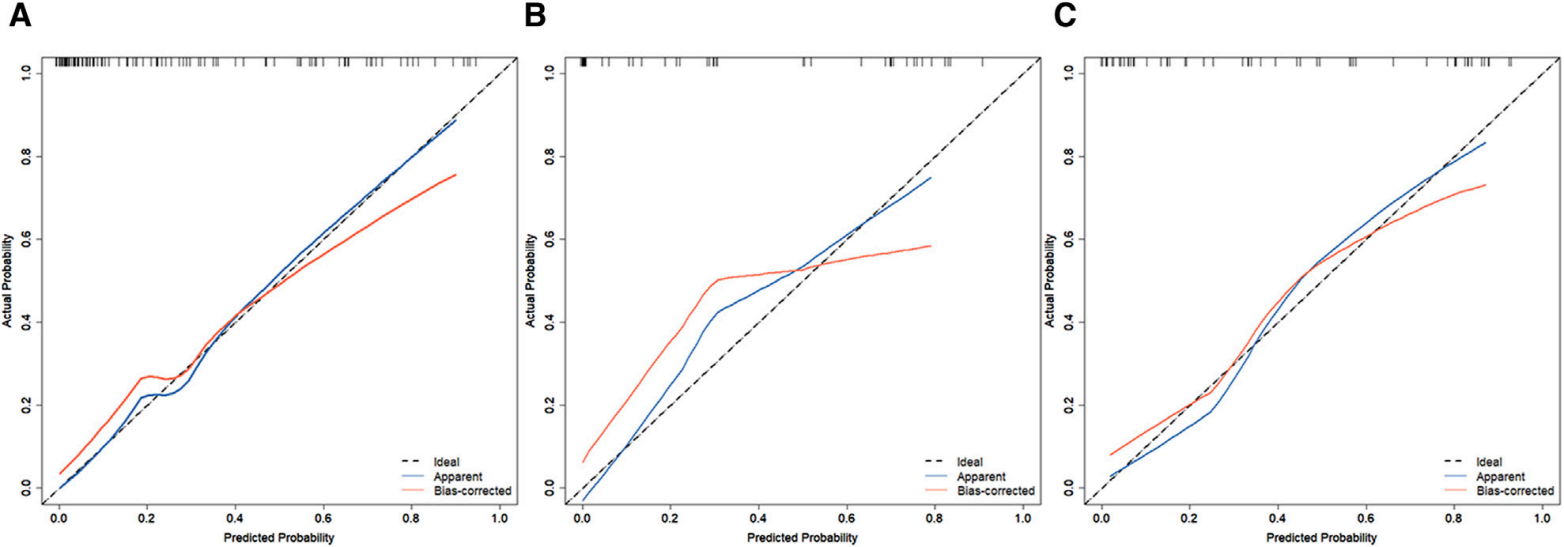
Calibration curves for predicting the probability of mortality in patients with CRO infections. **(A)** Training set. **(B)** Validation set. **(C)** External validation cohort.

**FIGURE 5:**
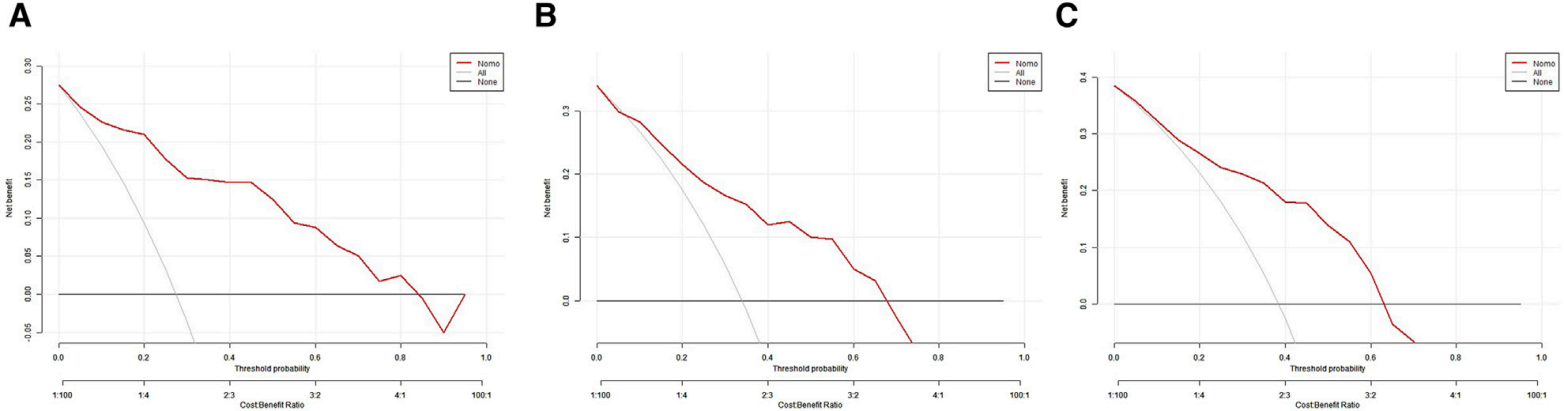
Decision curve analysis in prediction of mortality in patients with CRO infections. **(A)** Training set. **(B)** Validation set. **(C)** External validation cohort.

In the training set, the AUC was 0.888 (Figure 3A), and the calibration curve was close to the ideal diagonal line (Figure 4A). Furthermore, the DCA showed significantly better net benefit in the predictive model (Figure 5A). In addition, 65 patients from Xianning Central Hospital were used for external validation to test the nomogram. In the validation set and external validation cohort, the AUC was 0.799 (Figure 3B) and 0.805 (Figure 3C) respectively, reflecting a good accuracy of the nomogram. Meanwhile, the model had good consistency, and the calibration curves of the validation set and external validation cohort were also close to the ideal diagonal line (Figures 4B,C). Moreover, the DCA showed significant net benefit of the predictive model, as well as that in the validation cohort (Figures 5B,C). These results demonstrated that our nomogram has a significant potential for clinical decision-making.

## Discussion

Our study is the first to develop a model for predicting the occurrence of all-cause mortality of patients with CRO infections who underwent colistin sulfate treatment. While existing literature on colistin sulfate has predominantly centered on clinical efficacy, pharmacokinetics, and nephrotoxicity, our study delves into the prediction of mortality in this patient population. [(Wu et al., 2016; Geng et al., 2018; Bassetti et al., 2021; Zhang et al., 2024; Hao et al., 2022; Xie et al., 2022; Yu et al., 2022)] This study revealed that age, duration of combination therapy, nasogastric tube, previous surgery, polymicrobial infections, and septic shock were predictors of mortality in patients with CRO infections who underwent colistin sulfate treatment.

Postoperative mortality at 30 days has become a new quality measure in CRO infection. Several studies have shown that 28-day all-cause mortality remained high after treatment with colistin, including colistin sulfate. [(Jin et al., 2022; Wu et al., 2016; Geng et al., 2018; Bassetti et al., 2021; Zhang et al., 2024)-32] For this reason, the aim of this work was to develop a prediction model of 30-day all-cause mortality in a large series of CRO infected patients treated with colistin sulfate. Our study incorporated 170 patients in the study cohort, among whom 50 (29.4%) died within 30 days. This 30-day all-cause mortality rate is similar to those recently reported in other retrospective studies using domestic data sets (Jin et al., 2022; Peng et al., 2023; Huang et al., 2010).

When patients are treated with colistin sulfate-based regimens after CRO infection, we may be inclined to overlook and may not monitor the risks that may affect the benefit of treatment for CRO infection. Public health workers can use this nomogram to easily assess the risk of all-cause mortality in patients with CRO infection treated with colistin sulfate treatment. For high-risk specialty patients, this model can help clinicians to better collect high-risk information from patients, provide individualized pre-treatment education, and determining tailored prevention and management strategies.

In our risk prediction model, the independent risk factors associated with 30-day all-cause mortality were age, duration of combination therapy, nasogastric tube placement, previous surgery, polymicrobial infections, and septic shock. Many studies have shown that the duration of antimicrobial treatment is a key factor in the poor prognosis of CRO infection, and it is included in the standard treatment plan by the guidelines (Garnacho-Montero et al., 2017; Tamma et al., 2022; Zhang et al., 2022; Zhang et al., 2024). In our study, we recommend extending the duration of colistin sulfate treatment for CRO infection, and also recommend extending the duration of colistin sulfide-based combination therapy [*p* = 0.023; 95% CI = 0.032 (0.001–0.504)]. This recommendation is consistent with published research results. Because short-term treatments might only clear the microbiome, reduce ecological stress, and eliminate negative consequences (Tamma et al., 2017b; Albin et al., 2023), while long-term antibiotic treatment is necessary to avoid death due to repeated infection (Katip et al., 2023). At the same time, Insufficient treatment duration can elevate the risk of exacerbating other comorbidities.

Another two key factors associated with 30-day all-cause mortality was age and septic shock. Our study found that the risk of 30-day all-cause death treated with colistin sulfate was higher in CRO patients aged 40–60 years and over 60 years than in CRO patients aged under 40 years, [*p* = 0.030; 12.77 (1.573–170.3)] and [*p* = 0.076; 95% CI = 8.462 (0.898–109.4)]. Although *p* > 0.05 for patients aged over 60 years, age was still considered as a potentially important factor. This is consistent with the results of Zhang Haihui’s study. His research found that age was one of independent factors associated with all-cause mortality (Zhuang et al., 2023).

Our study also observed that septic shock was the predominant and significant predictor for 28-day mortality in our nomogram. As a clinical manifestation of the severity of infections and organ dysfunction, septic shock k has been shown to be associated with mortality in patients with CRO (Andria et al., 2015; Chumbita et al., 2022; Xiao et al., 2020; Zhang et al., 2020). Meanwhile, septic shock was also ranked as a significant and significant predictor of 28-day mortality in a study predicting 28-day all-cause mortality in patients with CRO (Jian et al., 2023).

It is worth noting that the presence of nasogastric tube is also a high-risk factor for mortality in patients with CRO infections [*p* = 0.002; 95% CI = 19.97 (3.617–178.8)]. This association may stem from the fact that patients with CRO infections often harbor comorbidities that compromise their immune function, leading to immune suppression and potential immune paralysis (Angus and Opal, 2016). Additionally, the necessity of a nasogastric tube, typically due to difficulties in oral intake, can contribute to malnutrition and exacerbate the immune deficiencies in patients, thereby complicating the management of CRO infections. This progression could potentially be linked to levels of albumin. In a prior study, we observed a direct correlation between CRO breakthrough infections and albumin levels in patients (Yang et al., 2023).

In this study, we assessed the infection-related factors influencing all-cause mortality in patients with CRO infections treated with colistin sulfate and built a risk prediction model for early prediction. Our external validation confirmed the good accuracy and conformity of the model, alongside its net benefit. This visual and personalized nomogram model provides clinicians with a simple and intuitive tool for practical prediction. Nonetheless, our study does have certain limitations. First, this was a retrospective cohort study with a limited number of enrolled patients, which affected the power of the test and may have led to the occurrence errors in the results. Second, the administration of colistin sulfate was a non-standardized regimen determined by the treating physician, which introduced bias. Therefore, there is a need to conduct larger, multicenter, prospective studies across a broader population range. By increasing the diversity of the sample and establishing strict dosing protocols, including dose, frequency, and duration of treatment, we can ensure that all patients receive treatment according to a unified standard. This will help to reduce bias due to sample size and non-standardized dosing regimens, thereby enhancing the quality and credibility of the research findings.

In conclusion, this study retrospectively analyzed infection-related data from the case database to identify fifteen risk factors linked to mortality in patients with CRO infections receiving colistin sulfate treatment. Through multivariate regression analysis, six independent risk factors were identified and used to develop a new nomogram with a reasonable level of accuracy. This tool can assist clinicians in assessing the risk of mortality in patients with CRO infections undergoing colistin sulfate therapy. By evaluating individual risk levels, healthcare providers can implement preemptive interventions to potentially extend patient survival.

## Data Availability

The raw data supporting the conclusions of this article will be made available by the authors, without undue reservation.
